# Kyphoplasty of a C2 Body Intraosseous Schwannoma: A Case Report

**DOI:** 10.7759/cureus.82264

**Published:** 2025-04-14

**Authors:** Michael Rodman, Diana Fang, Majid Khan, Qingqing Wu

**Affiliations:** 1 Neuroradiology, Johns Hopkins University School of Medicine, Baltimore, USA; 2 Pathology, Johns Hopkins University School of Medicine, Baltimore, USA

**Keywords:** antoni a, antoni b, intraosseous schwannoma, kyphoplasty, monoclonal gammopathy of undetermined significance, spinal intraosseous schwannoma, spinal schwannoma

## Abstract

We report a case of a 75-year-old male with an incidental and indeterminate lytic lesion of the C2 vertebral body. After rapid enlargement over 16 months, persistent neck pain, and concern for underlying occult malignancy, the patient underwent lesion biopsy and simultaneous balloon kyphoplasty. Histopathological analysis revealed the surprising diagnosis of intraosseous schwannoma. Spinal Intraosseous Schwannomas (SIS) are rare benign tumors, often mimicking more sinister processes on diagnostic imaging. Fortunately, their histologic appearance is identical to schwannomas found more commonly in the soft tissues, providing diagnostic certainty to otherwise ambiguous radiologic findings. Though rare, intraosseous schwannomas remain an important benign diagnostic consideration for incidental solitary lytic bone lesions. To our knowledge, this is the second published case of kyphoplasty involving an SIS.

## Introduction

Schwannomas are benign nerve sheath tumors arising from Schwann cells - the myelinating cells of the peripheral nervous system. These tumors can arise along any peripheral, cranial, or spinal nerve [1]. Pure intraosseous schwannomas are extremely rare and likely arise from the few nerve fibers normally traversing bony trabeculae, or from entrapped neural elements [2]. Due to their rarity and nonspecific imaging features, intraosseous schwannomas pose a radiologic diagnostic dilemma, and malignant entities must be excluded. When symptomatic, biopsy-proven intraosseous schwannomas are often treated with surgical resection, curettage and packing, or Stereotactic Body Radiation Therapy. Asymptomatic cases may be periodically monitored with imaging surveillance to assess for evidence of pathological fracturing or extraosseous involvement. In our case, the diagnosis of SIS was made post-kyphoplasty, which is a rather uncommon first-line therapy for this entity. 

## Case presentation

**Figure 1:**
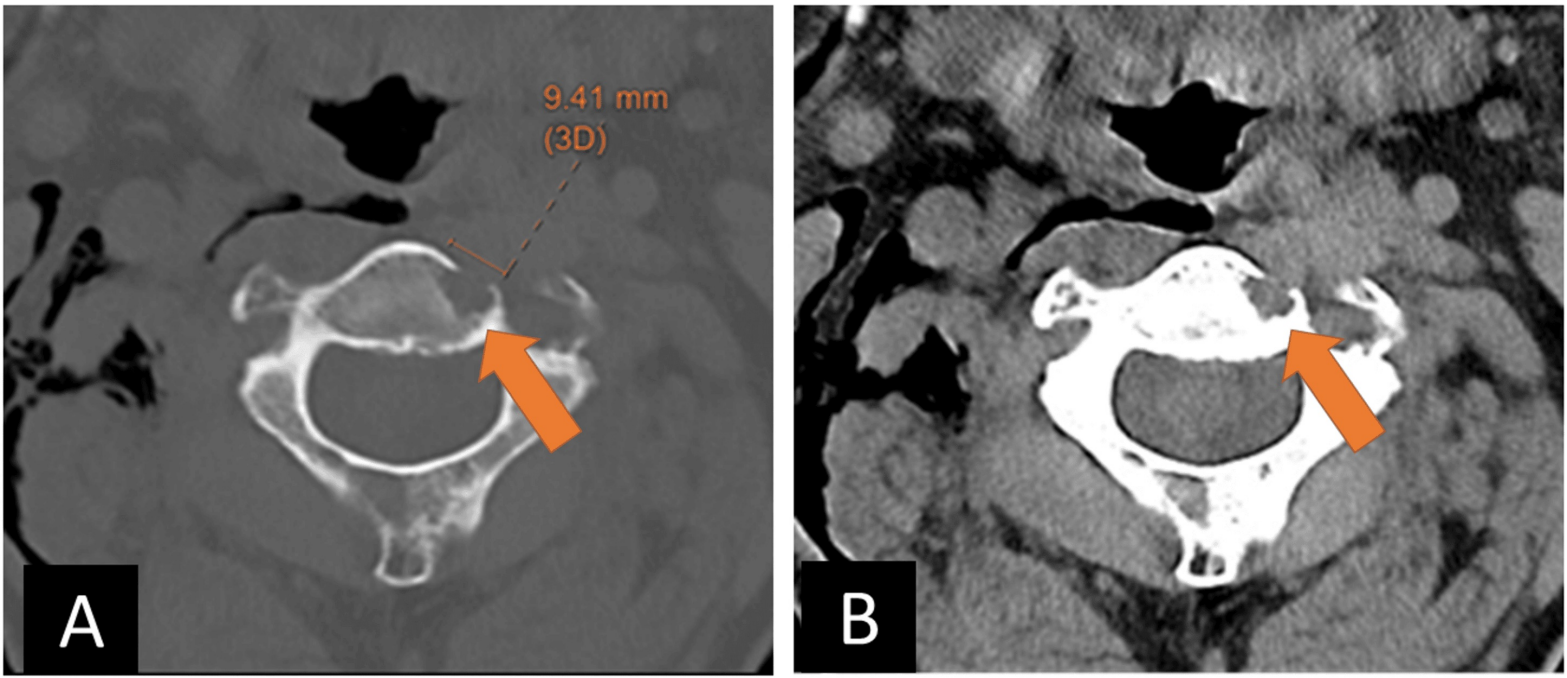
An incidental lytic lesion of the ventral C2 body with dehiscence of the anterior cortex, without appreciable soft tissue extension. Note subcutaneous emphysema of the right neck from trauma-related pneumothorax. A) axial bone window CT; B) axial soft tissue window CT.

A 75-year-old gentleman with a history of diabetic neuropathy, monoclonal gammopathy of undetermined significance, and heart failure initially presented to an outside emergency department after a fall. Noncontrast head and cervical spine CT demonstrated no acute findings in the neuraxis. An incidental subcentimeter lytic lesion of the ventral C2 body was noted, demonstrating a narrow zone of transition without peripheral sclerosis, bony scalloping, or periosteal reaction; there was dehiscence of the anterior C2 cortex. The lesion was dismissed as benign, and no differential diagnosis was given (Figure 1).

**Figure 2:**
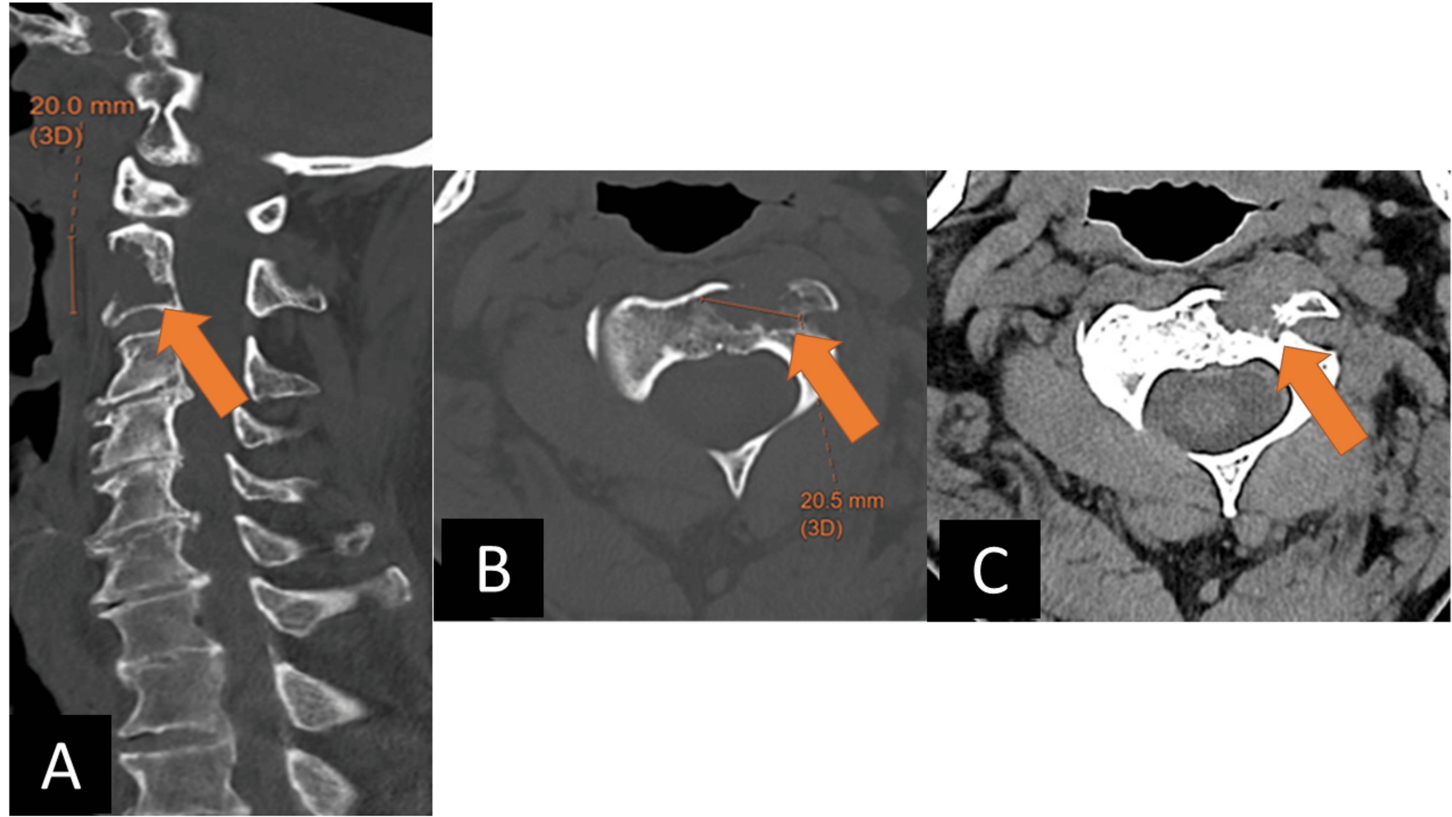
Repeat imaging of the cervical spine shows enlargement of the ventral C2 body lesion with progressed anterior cortical destruction, new involvement of the left foramen transversarium, and subtle extraosseous soft tissue anteriorly. A) sagittal bone window CT; B) axial bone window CT; C) axial soft tissue window CT.

Approximately 16 months later, the patient’s cervical spine was reimaged after a ground-level fall. The C2 vertebral body lesion showed substantial interval enlargement, with increasing cortical destruction - including of the left C2 foramen transversarium - and a small extraosseous component. Around this time, the patient endorsed generalized neck pain, though he exhibited a full range of motion of the neck and extremities. There was no muscle weakness or sensory deficit on physical exam (Figure 2). 

**Figure 3:**
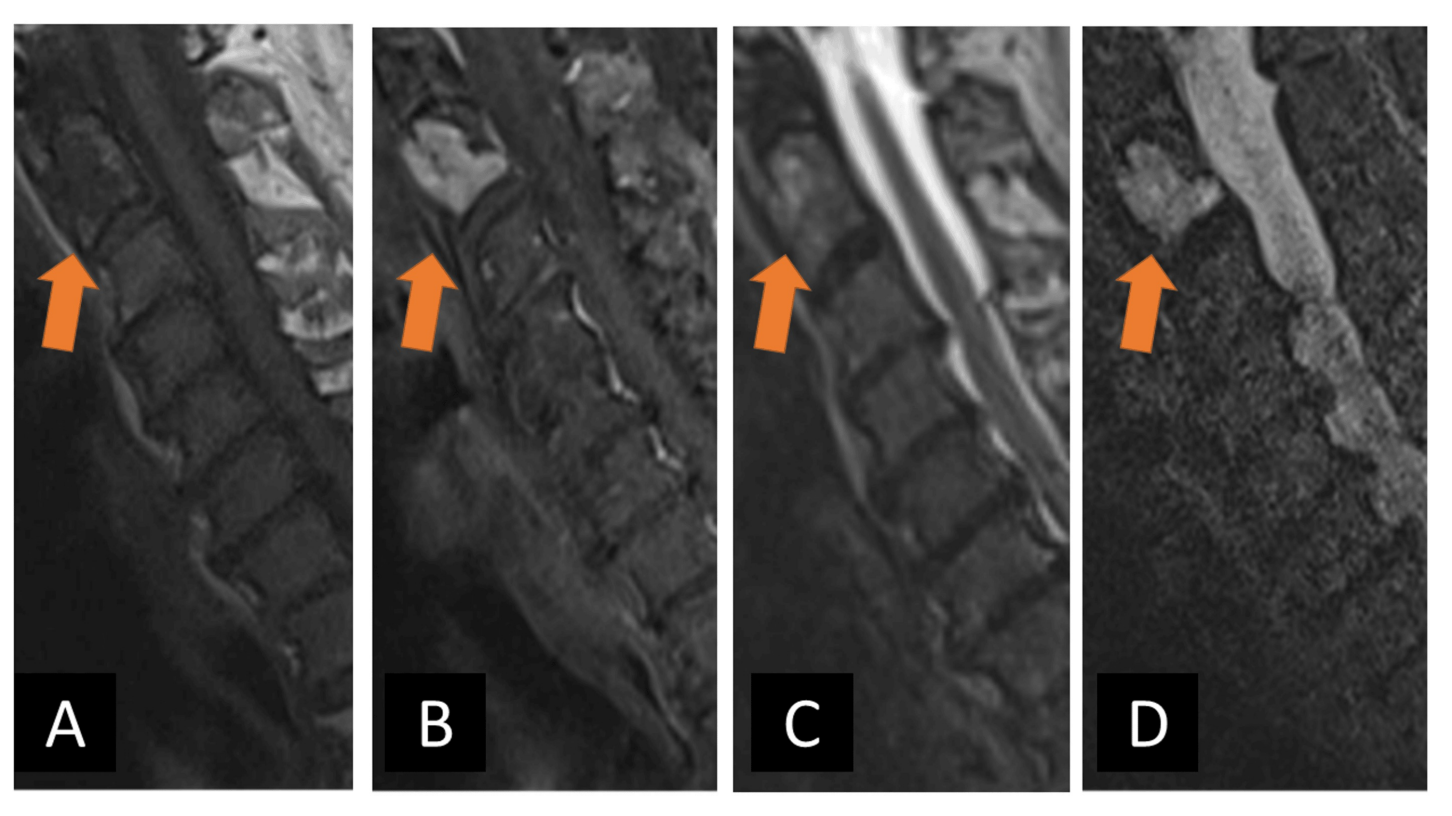
Cervical spine MRI shows a C2 lesion with anterior extraosseous extension (arrows). Cervical spine MRI shows a C2 lesion with anterior extraosseous extension (arrows) exhibiting A) low signal on non-contrast T1 weighted sequence, B) homogeneous contrast enhancement on post-contrast fat-saturated T1-weighted sequence, C) nearly isointense T2 signal, and D) hyperintense T2 STIR signal. There is no evidence of pathologic fracture. STIR: Short Tau Inversion Recovery

**Figure 4:**
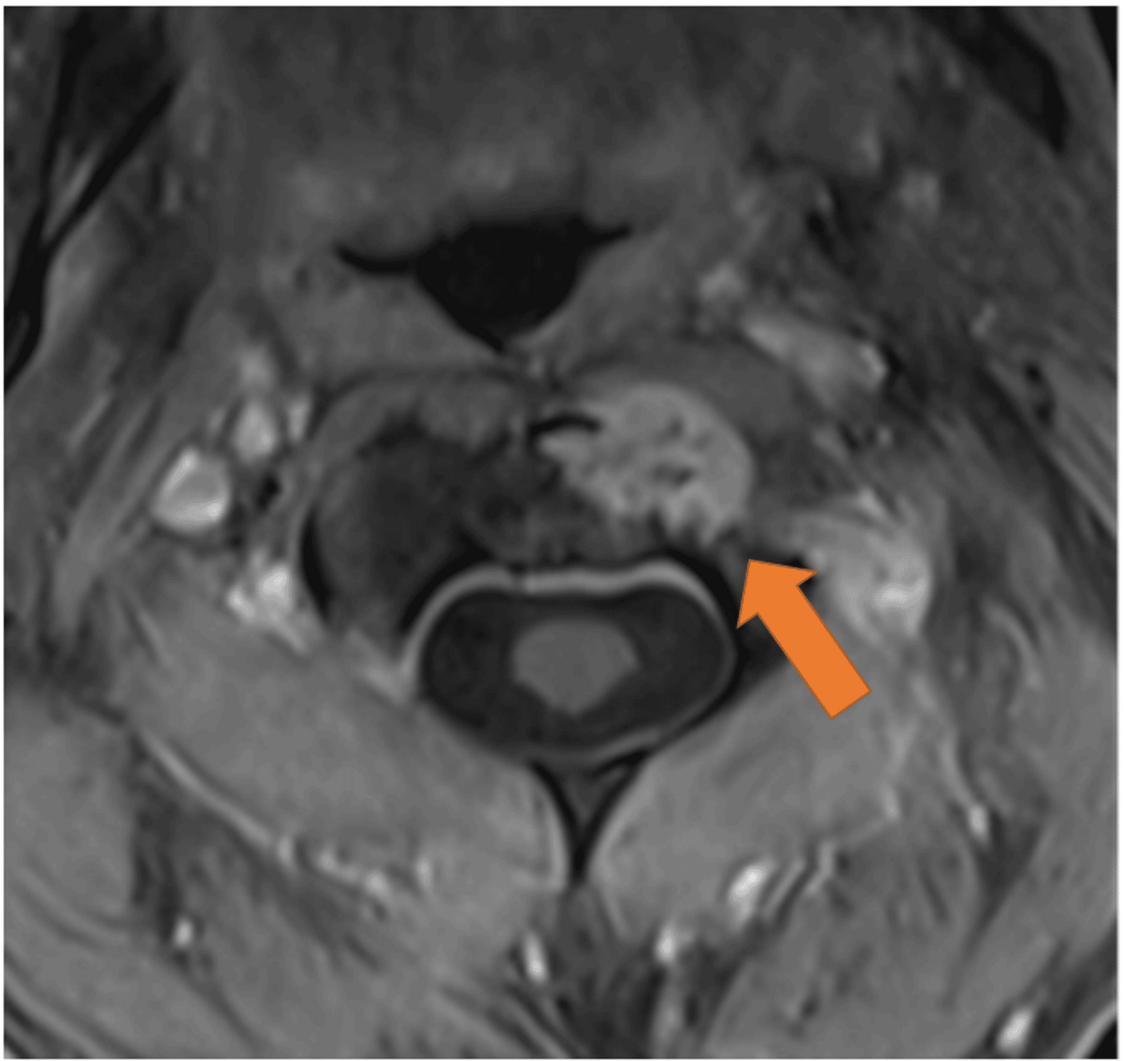
Post-contrast fat-saturated T1-weighted axial MRI image at the level of the C2 lesion shows anterior extraosseous disease uplifting the left longus colli muscle (arrow).

Due to the patient’s nonspecific neck pain, history of monoclonal gammopathy of undetermined significance, and the lesion’s growth, an MRI of the cervical, thoracic, and lumbar spine was obtained one month after the second fall. MRI imaging was notable for a solitary avidly enhancing T1 hypointense, T2 isointense-hyperintense, T2 STIR (Short Tau Inversion Recovery) hyperintense lesion at C2 with an extraosseous component uplifting the adjacent left longus colli muscle (Figures 3-4). There was no evidence of pathologic fracture or vertebral body collapse. Dynamic contrast enhancement and diffusion-weighted imaging were not obtained. The lesion was deemed indeterminate, and the differential included both benign and malignant entities. The possibility of a primary bone tumor such as chordoma or chondrosarcoma was raised, as these entities characteristically demonstrate bright T2 STIR signal and internal enhancement. Aggressive vertebral body hemangioma was also considered, though there was an absence of the stereotypical “polka dot” trabecular framework by CT. Additionally, solitary bone plasmacytomas often appear as destructive lytic lesions with similar T1 and T2 characteristics and variable enhancement. Multiple myeloma lesions render similar MR signal characteristics, and suspicion for this entity was high given the history of monoclonal gammopathy of undetermined significance; however, the lesion in question was an isolated finding. Finally, metastatic disease of unknown primary remained plausible. 

**Figure 5:**
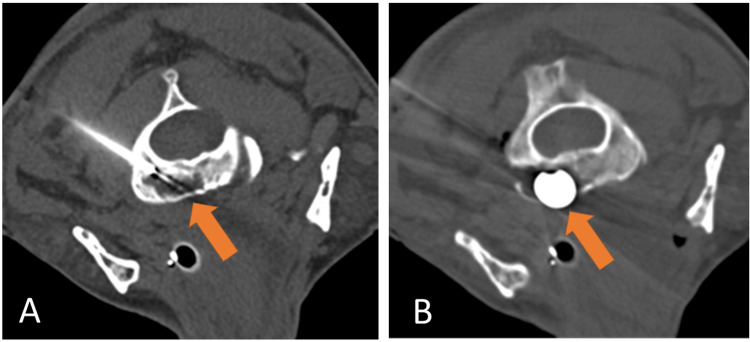
Intraoperative images during C2 lesion biopsy and kyphoplasty. A) axial bone window CT image showing posterior approach access of the C2 body with biopsy device deployed. B) axial bone window CT image showing balloon tamp reduction for cavity creation.

**Figure 6:**
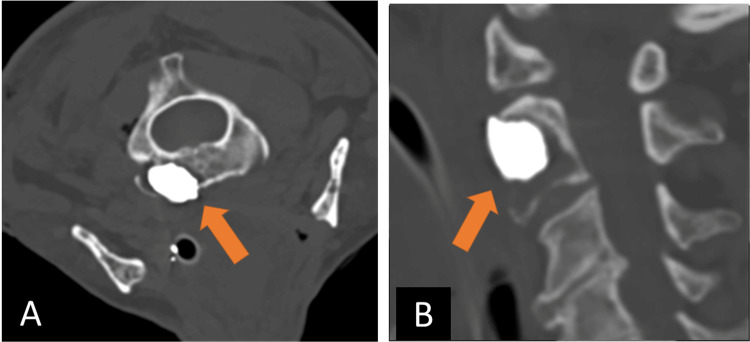
Intraoperative images during C2 lesion biopsy and kyphoplasty. Near complete filling of the lesion cavity with PMMA cement. (A) axial bone window CT; (B) sagittal bone window CT. PMMA: Polymethyl Methacrylate

After multidisciplinary discussion, the decision was made to percutaneously biopsy the lesion and simultaneously perform a balloon kyphoplasty. This approach was intended to stabilize the vertebral body from impending fracture and potentially alleviate the patient's neck pain. Approximately four months after the MRI, the patient was brought to the neurointerventional radiology suite and was intubated by a dedicated anesthesiology team. The patient was placed prone on the imaging table and a scout CT Angiogram from the skull base to the level of T5 was acquired for vessel mapping. The skin overlying the posterolateral neck was infiltrated with 1% preservative-free lidocaine. A 13G access cannula preloaded with a trochar-style tip was advanced to the base of the left C2 lamina via posterior approach and subsequently exchanged for a 13G hand drill. Access to the anterior C2 body was acquired under intermittent CT fluoroscopy with periodic drilling. Four 18G core biopsy samples of the bone lesion were acquired (Figure 5A). A 15 mm balloon was advanced into the biopsy cavity for kyphoplasty with Polymethyl Methacrylate (PMMA) cement (Figure 5B). The access cannula was removed. Postoperative CT imaging demonstrated near complete filling of the C2 lesion cavity and the access tract (Figure 6). The patient was extubated and short-term recovery was unremarkable. 

**Figure 7:**
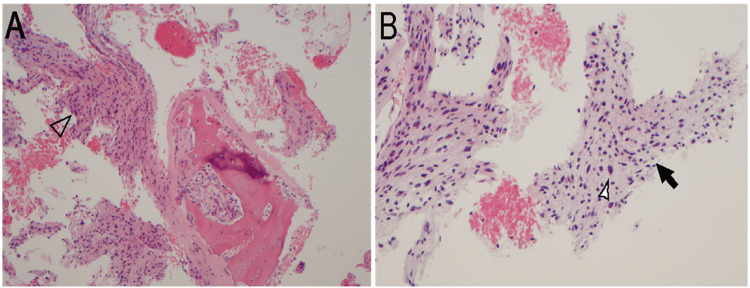
Intraosseous Schwannoma. A) neoplasm involving bone with Antoni A areas (arrowhead) on H&E at 100x magnification. B) neoplasm involving bone with Antoni B areas (black arrow) and scattered cells with degenerative atypia (arrowhead) on H&E at 200x magnification.

The biopsy showed a bland spindle cell neoplasm involving the bone. The neoplasm consisted of more compact areas with nuclear palisading (Antoni A) and slightly looser areas containing microcystic regions (Antoni B). Mitoses were inconspicuous and scattered cells exhibited degenerative atypia. The few vessels in the sample appeared hyalinized. The overall findings were consistent with an intraosseous schwannoma, an unexpected and benign entity (Figure 7). 

At a five-week follow-up clinic visit, the patient endorsed a near-complete resolution of his neck pain. With this reassuring clinical and histopathological information, the neurosurgery and radiology teams suggested preliminary surveillance with cervical spine MRI imaging at six-month intervals for two years; surgery or radiation therapy could be warranted if the lesion progresses or symptoms recur. 

## Discussion

Schwannomas are benign nerve sheath tumors that arise from Schwann cells of the peripheral nervous system, most commonly found in the lumbar spine and head and neck regions [1]. Spinal schwannomas often arise from spinal nerve roots as intradural extramedullary tumors and demonstrate transdural or extradural extension in about 30% of cases [2-4]. Rarely, schwannomas appear to arise from the bone itself and demonstrate invasive imaging features like osteolytic destruction. While intraosseous schwannomas account for less than 0.2% of all primary bone tumors, SIS are even more unusual and are often difficult to distinguish from malignant entities via diagnostic imaging [5]. 

The first intraosseous schwannoma was reported in the latter half of the 20th century, but numerous case reports have been published in the literature over the past 50 years. SISs arise at any spinal level, though the most commonly affected levels are unclear in the literature. A review of 20 SISs by Wang et al. indicates that the most common level involved is the cervical spine followed by the thoracic and lumbar spine [6]. A literature review of 24 cases by Zhang et al. shows near equal distribution of SIS in the cervical, thoracic, and lumbar spine [7].  

SIS can involve the bone by three suggested mechanisms: 1. Extraosseous tumor causing secondary bone erosion; 2. A tumor arising centrally within the bone; and 3. Tumor originating within the nutrient canal followed by growth into a dumbbell-shaped lesion [8-9]. The lesion in our case is remote from the neural foramina and spinal canal in its early stages, and only approaches the posterior C2 cortex after a year of growth, suggesting that the lesion arose centrally within the bone. Schwannomas arising primarily within the bone appear to be the most uncommon situation due to the scarcity of myelinated nerve fibers in bone; these nerves may arise from distal branches of the paired basivertebral nerves, which emanate from the sinuvertebral nerves [9]. The paucity of intraosseous nerve fibers likely accounts for the asymptomatic nature of many of these lesions [10]. While most patients are asymptomatic, potential symptoms include numbness, myelopathy, and focal pain localizing to the involved spinal level [6]. When large, the intraosseous schwannoma’s destructive nature can create bony instability that elevates the risk for pathologic fracture. 

After a discussion with our patient and doing an imaging review, the decision was made to stabilize the vertebral column with cementation at the time of biopsy. Hyperthermia from cement polymerization has been shown to elicit antineoplastic effects and damage adjacent nerves, which could alleviate any pain arising from the lesion [11]. Though schwannomas are exceedingly benign tumors and carry low rates of malignant transformation and recurrence, occasional follow-up imaging will be required to assess for lesional growth in our case as the lesion was not completely resected or curettaged [4]. Our treatment teams decided on initial surveillance MRI imaging every six months for two years postoperatively. Fortunately, our patient will be excused from further oncologic workup with regard to this spinal neoplasm. To our knowledge, this is the second published case of kyphoplasty involving a spinal schwannoma, referencing a recent case report by Rahyussalim et al., in which the histologic diagnosis was made preoperatively [12]. 

The radiological appearance of SIS overlaps with several benign and malignant entities. For example, chordomas and chondrosarcomas can show aggressive lytic bone changes with hyperintense T2 signal and variable enhancement [13-14]. Aggressive spinal hemangiomas also demonstrate T2 hyperintense signal, avid enhancement, and destructive osseous changes, but typically show accentuated internal trabecular framework [15]. Solitary bone plasmacytomas classically demonstrate a “cerebriform” matrix and T1 signal slightly above that of skeletal muscle; in the case of an isolated enhancing vertebral body tumor, this is an important consideration [16]. Finally, metastatic disease of unknown primary and multiple myeloma were important considerations in our patient with monoclonal gammopathy of undetermined significance, though MRI imaging demonstrated that the C2 lesion was an isolated finding [17]. In any case, a biopsy is imperative for a definitive diagnosis.

## Conclusions

Here we report a case of an incidental Spinal Intraosseous Schwannoma, a rare differential consideration for a primary vertebral body tumor. Imaging features of SIS demonstrate similarities with both benign and malignant entities, and thus tissue sampling is imperative to exclude malignant disease and guide patient management. To our knowledge, this is the second report of an SIS treated with balloon kyphoplasty and cementation, which preceded an unexpectedly benign histopathological diagnosis.
